# SARS-CoV-2 Infection and Clinical Signs in Cats and Dogs from Confirmed Positive Households in Germany

**DOI:** 10.3390/v15040837

**Published:** 2023-03-24

**Authors:** Anna Michelitsch, Valerie Allendorf, Franz Josef Conraths, Jörn Gethmann, Jana Schulz, Kerstin Wernike, Nicolai Denzin

**Affiliations:** 1 Institute of Epidemiology, Friedrich-Loeffler-Institut, 17493 Greifswald, Germany; 2 Institute of International Animal Health/One Health, Friedrich-Loeffler-Institut, 17493 Greifswald, Germany; 3 Institute of Diagnostic Virology, Friedrich-Loeffler-Institut, 17493 Greifswald, Germany

**Keywords:** coronavirus, companion animals, COVID-19, risk assessment, zooanthroponosis

## Abstract

On a global scale, the severe acute respiratory syndrome coronavirus 2 (SARS-CoV-2) poses a serious threat to the health of the human population. Not only humans can be infected, but also their companion animals. The antibody status of 115 cats and 170 dogs, originating from 177 German households known to have been SARS-CoV-2 positive, was determined by enzyme-linked immunosorbent assay (ELISA), and the results were combined with information gathered from a questionnaire that was completed by the owner(s) of the animals. The true seroprevalences of SARS-CoV-2 among cats and dogs were 42.5% (95% CI 33.5–51.9) and 56.8% (95% CI 49.1–64.4), respectively. In a multivariable logistic regression accounting for data clustered in households, for cats, the number of infected humans in the household and an above-average contact intensity turned out to be significant risk factors; contact with humans outside the household was a protective factor. For dogs, on the contrary, contact outside the household was a risk factor, and reduced contact, once the human infection was known, was a significant protective factor. No significant association was found between reported clinical signs in animals and their antibody status, and no spatial clustering of positive test results was identified.

## 1. Introduction

Ever since the World Health Organization (WHO) declared the outbreak of the severe acute respiratory syndrome coronavirus (SARS-CoV-2) in the city of Wuhan, China, as a public health emergency of international concern in January 2020 [1] and as a pandemic later on in March 2020 [2], the role of various animals in the origin, transmission, and evolution of SARS-CoV-2 was a subject of debate [3]. In particular, domestic cats (*Felis catus*), as well as dogs (*Canis lupus familiaris*), were at the center point of the discussion since they are an essential part of the lives of many humans worldwide [4]. A global survey estimated that 57% of people own at least one pet animal. Dogs lead the chart at 33%, followed by cats at 29% [5]. Since both domestic animal species not only proved to be susceptible to infection with SARS-CoV-2 in early animal trials [6,7,8,9] but also showed a regular occurrence of natural infections in various prevalence studies around the world [10,11,12,13,14,15,16,17,18,19,20,21,22,23], the question arose of what the consequences of an infection with SARS-CoV-2 are not only for the animals themselves but also for the households and in a broader context, the direct environment of the infected animal.

Although the occurrence of a SARS-CoV-2 infection in both cats and dogs has frequently been reported, there is presently little evidence that these animals play an active part in the transmission dynamic [3,24]. Other than a suspected transmission from an infected cat to the attending veterinarian [25], no case of interspecies transmission from these two animal species to humans has been documented to date. However, the possibility of such an animal-to-human transmission cannot be ignored since it has already been shown for other animal species. The first instance occurred in a fur production facility between farm workers and mink kept for fur production [26]. This was followed by multiple outbreaks in mink farms in Europe [26,27,28,29]. Further, an outbreak among humans in Hong Kong was linked to a pet shop selling Syrian hamsters [30,31]. In these settings, the risk of the evolution of a new variant is present, as was seen during an outbreak in a Danish mink farm that led to a cluster of human infections [27,28]. Although the mutations acquired in mink-associated SARS-CoV-2 virus isolates, such as Y453F, seem to attenuate the virus in human airway cells [32], the evolution of the virus in such a setting is hard to predict and needs constant surveillance [33].

In order to acquire a deeper understanding of the role of cats and dogs in the transmission dynamics of SARS-CoV-2, an epidemiological study was conducted. The primary aim of the study was to obtain an explorative data set for the evaluation of occurrence, impact, and implications of SARS-CoV-2 infection in cats and dogs kept in households with confirmed human infection. Additionally, some background information concerning the course of infection in humans was collected. Households that were known to be SARS-CoV-2 positive were asked to participate with their cats and dogs. The combination of the determination of the serological status of the animals with an individually answered questionnaire that was filled out by the respective owner(s) of the animals, enabled an analysis of risk factors for interspecies transmission and possible signs of disease after SARS-CoV-2 infection in cats and dogs.

## 2. Materials and Methods

Participation in the study was granted if prerequisites (see below) were met. From each animal that took part in the study, a single serum sample was taken. In addition, a questionnaire (Questionnaire S1) concerning general data, observed clinical signs, and human–animal interaction had to be completed for each animal and submitted by the respective owner(s). Only complete data sets containing both the serum sample and the completed questionnaire were included in the final analysis.

Participating households had to have at least one case of PCR-confirmed SARS-CoV-2 infection in any household member within three months prior to the date of sampling and at least one pet (cat or dog) that they were willing to let take part in the study. The owner(s) of the animals were informed about the purpose of the study, the sampling, and data collection procedures. They had to consent to a declaration of readiness for participation in the study as well as a declaration of consent under data protection law according to Art. 4 No. 11, 7 EU-DSGVO. The costs of the blood sampling at the local veterinarian were incurred and the owner(s) were additionally awarded compensation for their effort of EUR 35 per animal.

Participants were acquired through convenience sampling, meaning that everyone who applied for participation and fulfilled the aforementioned criteria was allowed to take part with any number of pets (i.e., cats and dogs) living in the household. Since the targeted population was hard to reach, an approach best described as indirect snowball sampling [34] was chosen. Attention to the study was generated through two approaches. Firstly, the Chambers of Veterinarians in each German federal state were asked to alert the veterinary practices and clinics of their state to the study and ask them to display an information sheet in their premises. All veterinary practitioners are members of a Chamber of Veterinarians in Germany. This approach provided the additional benefit of assuring veterinarians of the legitimation of the study when participating pet owner(s) asked them for the sample collection within the frame of the study. The 17 federal veterinary chambers offered different degrees of support. Ten chambers answered to the formal request sent via e-mail. Eight of them sent a corresponding note to their members and one released a note on their homepage. Second, an online approach was chosen. Therefore, various online platforms aimed at pet owners and veterinarians were contacted and asked for support in promoting the study. This resulted not only in notices on multiple homepages but also in a variety of posts on popular social media platforms that spread through sharing in the targeted community. This virtual snowball sampling [35] was further amplified by word of mouth, meaning that participants told their friends and neighbors about the study.

A single serum sample was taken from each participating animal by the family veterinarian during a routine health check and sent to the Friedrich Loeffler Institute (FLI), Federal Research Institute for Animal Health of Germany, for testing. Since sampling was performed in the context of a diagnostic test, no ethical approval was needed in consultation with the relevant state ethics committee (State Office for Agriculture, Food Safety and Fishery in Mecklenburg-Western Pomerania). Accordingly, owners were informed about the test results that were obtained for their pets. Participants were asked to aim for an appointment approximately four weeks after infection was confirmed by PCR test in at least one human of the respective household. However, a timeframe between three weeks and at most three months was allowed for inclusion in the study. This slot was chosen based on the sparsely existing data on the course of antibody titres naturally infected cats [10,36].

Serum samples were tested for the occurrence of antibodies against SARS-CoV-2 by a validated indirect multispecies ELISA [37]. The ELISA was based on the receptor-binding domain (RBD) of the SARS-CoV-2 spike protein [37]. In brief, 50 µL of a 1/100 dilution of each serum sample in Tris-buffered saline, pH 7.4, with Tween 20 (TBST) was incubated simultaneously in a well coated with the RBD of the SARS-CoV-2 spike protein and an uncoated well in microplates for one hour. After a washing step with TBST, all wells were incubated with a multispecies conjugate (SBVMILK; Innovative Diagnostics, Grabels, France). After another hour, a second washing step was performed and tetramethylbenzidine (TMB) substrate (IDEXX GmbH, Kornwestheim, Germany) added to the wells. The stop solution (IDEXX GmbH, Kornwestheim, Germany) was added after ten min and reading was performed at a wavelength of 450 nm on a Tecan Infinite M200 Pro microplate reader (Tecan Group Ltd., Männedorf, Switzerland). By subtraction of the optical density (OD) value of the uncoated well from the OD value of the coated well, the absorbance was determined. An OD of ≥0.3 was defined as positive, while smaller values were declared as negative [37]. The intermediate zone of the test, which is defined as the values between 0.3 and 0.2, was declared as negative, since the sampling occurred in a period where high antibody values were expected.

Owners were asked to complete a questionnaire for their participating animals. Inquiries involving understanding or interpretation of the questions that occurred, although the questions were formulated as clearly as possible, were not answered. Instead owners were asked to answer in accordance with their own understanding in order to avoid influence on the answer given [38].

Questions were either dichotomous, yielding dichotomous data, or they were dichotomized for univariable and partly multivariable analysis as follows. The interaction between animals and owner(s) was queried on a multi-item psychometric scale [39]. For analysis, a four-point rating scale was coded as follows: ‘daily’ (4), ‘several times a week’ (3), ‘sometimes’ (2), ‘never’ (1). An interaction score was calculated by building the sum of the values of all questions concerning the interaction. The mean value was then calculated for the interaction scores of all participants. Participants with an interaction score above the mean were classified as having a high interaction and participants with an interaction score below the mean were classified as having low interaction.

Clinical signs of the animals were queried in a nominal-polytomous question for the time before, during, and/or after quarantine of the household. The information was unified for all three timepoints. Further, clinical signs of the respective animal were grouped according to the organ system affected. Therefore, the given options ‘cough’, ‘nasal discharge’, and ‘laboured breathing’ were grouped as ‘respiratory symptoms’. ‘Reduced resilience’ as well as ‘reduced appetite’ and ‘increased need for rest’ were combined as ‘reduced general health’. ‘Diarrhea’ was classified as ‘gastrointestinal symptoms’. Further, answers given under the free-text option ‘other symptoms’ were grouped into one of the described groups according to their affiliation.

Reported chronic diseases were also dichotomized as ‘one or more chronic disease reported’ and ‘no chronic disease reported’ since the reported individual diseases were too diverse with very low respective frequencies to warrant meaningful analysis.

Background information concerning the course of infection in humans was evaluated descriptively, with data referring to infection in pets descriptively and analytically.

Statistical analysis was performed in order to identify factors influencing the risk of infection of pet animals and associations of clinical signs with seropositivity. Analysis was performed separately for cats and dogs, since pre-analyses revealed differences in the effect of potentially influential factors between these species. An unifactorial analysis was performed by using univariable generalized logistic regression models with binomial error distribution for each factor. For univariable analysis only, the following data were dichotomized. Reported age as well as weight were dichotomized into low and high, applying the respective mean as the threshold for dogs and cats. The data concerning the number of infected humans were split into the categories of one and more than one infected human. Further, the data retrieved from the closed-ended trichotomous question concerning the change of interaction between animals and owner(s) were dichotomized by combining the responses ‘yes, reduced’ and ‘yes, discontinued’ to ‘yes’ in contrast to ‘no, everything remained unchanged’. Factors with a *p*-value of lower than 0.2 were used for a multivariable logistic regression model [40]. In this multivariable analysis, the reported number of infected humans (n) per household were used as numerical data and the data retrieved from the question concerning the change of interaction between animals and owner(s) were used in an ordinal form. The model was then optimized by stepwise backward reduction using the Akaike information criterion (AIC) [41]. Both univariable and multivariable analysis were corrected for clustering among households through calculation of robust standard errors [42]. A *p*-value of 0.05 or lower was assumed as indicative of a significant association.

To analyze the situation in multiple-pet households, a data subset was created that contained only households with more than one participating animal. Pets that lived in a household with at least one seropositive animal were classified as having other seropositive animals in the household, and again, a univariable generalized regression model was calculated.

Further, the correlation between participating households and animals, respectively, and inhabitants per federal state was analyzed by a linear regression model and the geographic origin of the samples was displayed employing a Geographic Information System (GIS; Karten-Explorer©, version 2.21). In addition, a spatial scan statistic (SaTScan™, version v10.1, [43]) was used to assess potential spatial clustering. In this analysis, the positive test samples (cats and dogs combined) represented the cases, and negative test samples the controls. Therefore, the Bernoulli model was chosen when running the scan statistic. As recommended [44], the maximum window size was set to capture up to 50% of the events (cases and controls).

Reported clinical signs were analyzed by a univariable generalized logistic regression model with binomial error distribution for each symptom category separately for cats and dogs.

The confidence intervals for the apparent and true seroprevalences in dogs and cats of infected households were calculated using the Clopper–Pearson [45] method. The true prevalence was calculated according to Rogan and Gladen [46] based on the sensitivity (98.31%) and specificity (100%) reported for the test in use [37].

Statistical analysis was conducted in R [47] with packages sandwich [48,49] and lmtest [50]. Figures were created using the packages ggplot2 [51] and UpSetR [52,53].

## 3. Results

### 3.1. Properties of Participating Households and Respective Human and Companion Animal Members

**Figure 1:**
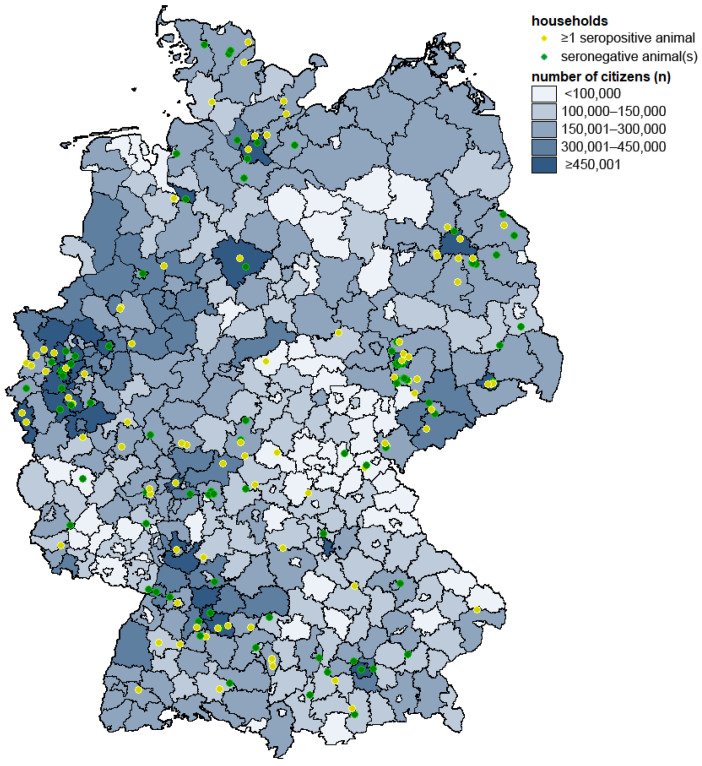
Geographic origin of serum samples in Germany. Households with at least one seropositive pet (cat or dog) sample are represented by yellow dots and households with only seronegative samples by green dots. The number of human inhabitants living in each district is given in shades of blue according to the legend.

**Figure 2:**
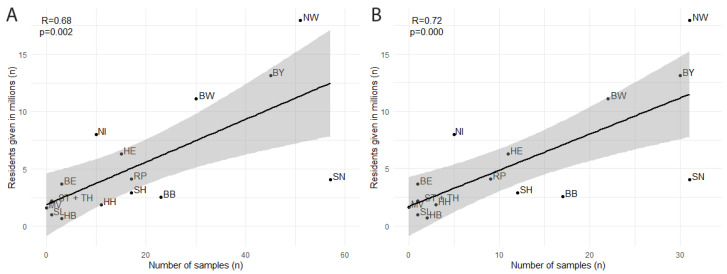
Univariable linear regression model of participants (**A**) as well as participating households (**B**) from each German federal state in dependence of human inhabitants given in millions. Line fits represent linear regression and 95% confidence interval. R = Spearman correlation coefficient, *p* = associated *p*-value, BB = Brandenburg, BE = Berlin, BW = Baden-Württemberg, BY = Bavaria, HE = Hesse, HB = Bremen, HH = Hamburg, MV = Mecklenburg-Western Pomerania, NI = Lower Saxony, NW = North Rhine-Westphalia, RP = Rhineland-Palatinate, SH = Schleswig-Holstein, SL = Saarland, SN = Saxony, ST = Saxony-Anhalt, TH = Thuringia.

Overall, 285 animals, 115 cats and 170 dogs, from 177 households participated in the study. The initial confirmation of an infected human household member via PCR occurred from September to December 2021. The mean time interval from the aforementioned confirmation of human infection in the household until blood sampling of the participating animal was 38.0 ± 14.6 (mean ± standard deviation) days. Participating households were located all over Germany (Figure 1). The number of households and individual animals acquired for the study was significantly correlated (*p* = 0.002 and *p* < 0.001, respectively) with the human population of each federal state [54] (Figure 2). No spatial clustering of positive cases was identified.

The mean number of human household members was 2.9 ± 1.3, from which a mean of 2.0 ± 1.5 members was infected with SARS-CoV-2. The majority of households reported at least one human member showing symptoms during SARS-CoV-2 infection (*n* = 176, 99.4%). Between the first positive PCR test and the onset of symptoms, 0.1 ± 7.1 days passed by. The most common symptoms in humans were having a cold (*n* = 157, 88.7%), coughing (*n* = 148, 83.6%), headache (*n* = 133, 75.1%), loss of smell or taste (*n* = 132, 74.6%), and having a sore throat (*n* = 104, 58.8%). When asked about other symptoms, the most common statement was some kind of pain in the limbs or joints (*n* = 27, 15.3%).

**Figure 3:**
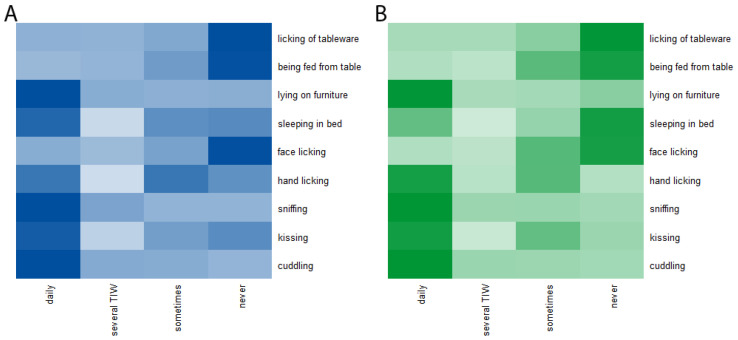
Heatmap of interactions between cats (**A**) as well as dogs (**B**) and their owner(s). TIW = times a week.

Half of the participating animals were male (*n* = 144, 50.5%). Approximately one-third of dogs (*n* = 59, 34.7%) were described as mixed breed. The most frequent dog breeds reported were the Labrador Retriever (*n* = 12, 7.1%), the French Bulldog (*n* = 10, 5.9%), and the Border Collie (*n* = 9, 5.3%). Cats were predominately described as mixed breed resp. as ‘European Shorthair’ being a synonym for an unspecified origin (*n* = 82, 71.3%). The British Shorthair was the most frequently reported cat breed (*n* = 12, 10.5%) followed by the Ragdoll (*n* = 5, 4.3%). One-quarter of the participating animals were reported to have one or more chronic diseases (*n* = 73, 25.6%). Further, 79 (27.7%) had regular unsupervised outdoor access and 82 (28.8%) owners stated that they reduced or stopped interaction with their animal after diagnosis of infection with SARS-CoV-2. The most frequent interaction between the owner(s) and the animal that occurred on a daily basis was cuddling (*n* = 249, 87.4%), followed by letting the animal sniff (hands, face) (*n* = 214, 75.1%), the allowance to lay on furniture (*n* = 194, 68.1%), and kissing the animal (*n* = 119, 41.8%). The allowance to lick used tableware (*n* = 8, 2.8%), feeding the animal from the table (*n* = 16, 5.6%), letting the animal lick the owners face (*n* = 29, 10.2%), to sleep on the owners’ bed (*n* = 88, 30.9%) as well as letting the animal lick the hands (*n* = 112, 39.3%) were the least frequent interactions that occurred on a daily basis (Figure 3).

**Figure 4:**
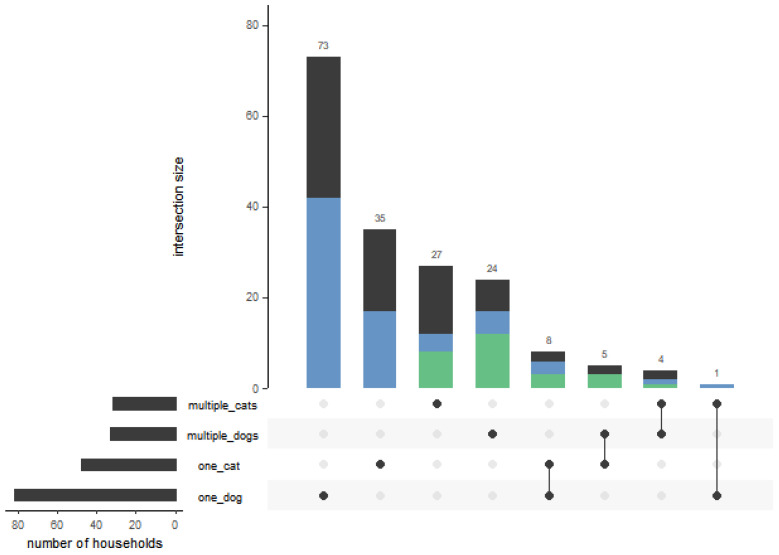
Structure of the pet holdings participating in the study. The vertical columns represent the number of households that own animals of a specific category or resp. category combination as indicated by black dot(s) below. The black lines interlinking the black dots indicate respective combinations of categories in the household. Households with at least one positive animal are marked in blue; households with at least two positive animals are marked in green. The horizontal columns on the left-hand side represent the sum of all households sharing the indicated category.

Approximately half of the households (*n* = 83, 46.9%) stated that there was more than one pet animal in the household. A total of 69 households (39.0%) actually participated with more than one animal and 44 (63.8%) of the latter housed at least one positive animal. In 30 (43.5%) of the multiple-pet households, more than one animal was seropositive (Figure 4). Of these 30 households, all animals of the household of which samples were submitted were seropositive in 22 (73.3%) households. Interestingly, in five of the eight households in which not all participating animals acquired an infection, the animal that remained testing negatively was the only animal in the respective household with a lower-than-average interaction score. Of the 177 animals living in multiple-pet households, 94 (53.1%) had another positive animal in their households.

Most participating animals were reported to have been asymptomatic before, during and after human quarantine (*n* = 166, 58.3%). Of the 119 animals that showed clinical signs, 22 experienced them before quarantine, 96 during, and 59 after the end of the quarantine. The most common symptom described was an increased need for rest (*n* = 55, 19.3%), followed by diarrhea (*n* = 45, 15.8%), a reduced appetite (*n* = 36, 12.6%), nasal discharge (*n* = 27, 9.5%), coughing (*n* = 26, 9.1%), labored breathing (*n* = 21, 7.4%), and reduced resilience (*n* = 21, 7.4%). Additional symptoms to the one specifically asked for in the questionnaire were reported for 48 (16.8%) animals with sneezing being the most frequent (*n* = 22, 7.7%). Grouping for statistical analysis found that of the 119 (41.8%) animals that showed clinical signs at some point during quarantine, 74 (26.0%) experienced a reduction in general health, 62 (21.8%) respiratory symptoms and 49 (17.2%) gastrointestinal symptoms.

For further details of the categorized information separated according to animal species, see Table 1 for parameters and Table 2 for reported clinical signs.

### 3.2. Seroprevalence in Dogs and Cats

One-hundred-and-forty-three (50.2%) samples tested positive in the indirect ELISA. A total of 48 (41.7%) of all cat sera and 95 (55.9%) of all dog sera tested positive. This leads to an apparent prevalence of 41.7% (95% CI 32.6–51.3) for cats and of 55.9% (95% CI 48.1–63.5) for dogs. Further, the true prevalence for cats was calculated as 42.5% (95% CI 33.5–51.9) and 56.8% (95% CI 49.1–64.4) for dogs. Details about absorbance can be found in the supplements (Figure S1).

### 3.3. Risk Factors for Infection and Clinical Signs

#### 3.3.1. Univariable Statistical Analysis

The determined seroprevalence of dogs was higher than the seroprevalence in cats (1.77 OR 95% CI 1.00–3.13, *p* = 0.051) but not significant at the specified significance level of 0.05.

The factor of more than one infected human in a household was significantly associated with the probability of a cat testing positive for antibodies to SARS-CoV-2 (3.08 OR 95% CI 1.11–8.54). Further, univariable analysis revealed a trend for seropositive cats to be less likely to have unsupervised outdoor access (0.45 OR 95% CI 0.18–1.13) as well as contact with humans outside the household (0.31 OR 95% CI 0.06–1.45) and a higher contact intensity with their owner(s) (2.32 OR 95% CI 0.96–5.62). Seropositive dogs showed a trend of being more likely to have contact with humans outside the household (1.99 OR 95% CI 0.89–4.45). Further, there was a trend that their owner(s) were less likely to reduce or stop contact during quarantine (0.45 OR 95% CI 0.17–1.18) and that more than one human of the household was infected (2.15 OR 95% CI 0.95–4.84). Consistent with findings in humans, the sex ‘male’ and a higher age increased the probability of both cats and dogs to test positive, but the association was not significant. For details see Table 3.

In households with more than one participating animal, seropositive animals were significantly more likely to have another positive animal in the household than seronegative animals (9.63 OR 95% CI 3.69–25.16, *p* < 0.001).

The categorized reported clinical signs did not show significant differences between seropositive and seronegative cats and dogs, respectively. For details, see Table 4.

#### 3.3.2. Multivariable Statistical Analysis

Stepwise backward reduction of the logistic regression model for cats reduced the AIC from a starting value of 145.19 to a value of 143.80 in the final model. The factors that remained in the final optimized regression model for cats were the numerical factor ‘infected humans per household’ as well as the binominal factors ‘above average contact intensity’, ‘unsupervised outdoor access’, and ‘contact outside the household’. More infected humans in the household were significantly associated with the probability of a cat being positive for antibodies to SARS-CoV-2 (2.00 OR 95% CI 1.38–2.91; *p* < 0.001). Further, seropositive cats were significantly more likely to have an above-average contact intensity (2.54 OR 95% CI 1.10–5.85; *p* = 0.03).

**Figure 5:**
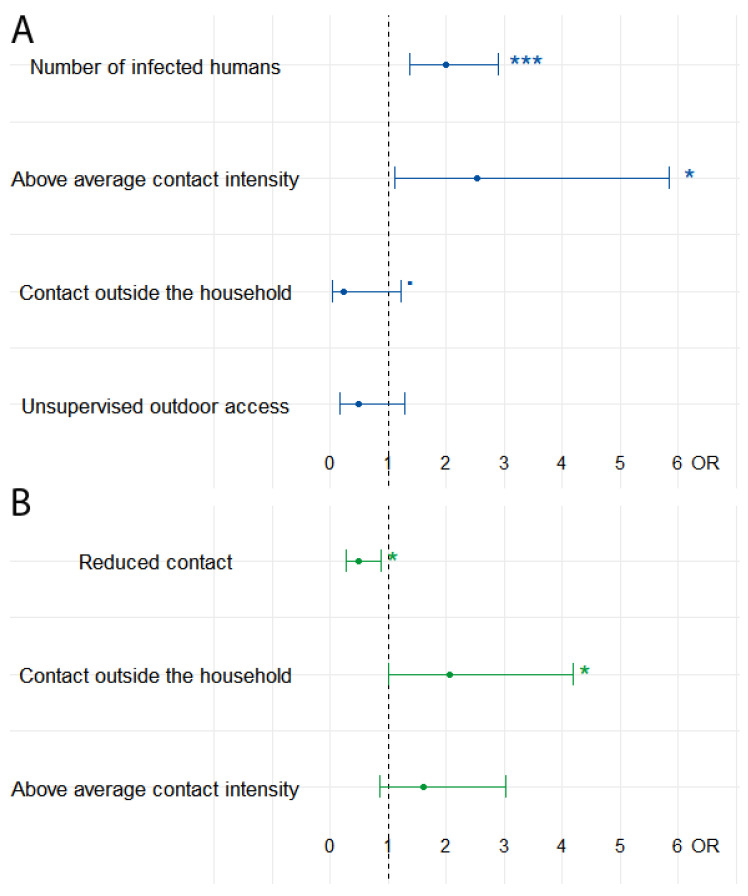
Optimized multivariable regression models for cats (**A**) and dogs (**B**). *p*-values are categorized as follows: *** < 0.001; * < 0.05; ▪ < 0.1.

The optimization of the logistic regression model for dogs reduced a starting AIC of 232.08 to 228.5 in the final model. The factors that were included in the final optimized regression model were the binominal factors ‘contact outside the household’ and ‘above average contact intensity’ as well as the ordinal factor ‘contact reduction’. Dogs were significantly less likely to turn out seropositive when the owner(s) reduced contact with their animal during quarantine (0.49 OR 95% CI 0.27–0.87; *p* = 0.02). Further, seropositive dogs were significantly more likely to have contact with a human outside the household (2.05 OR 95% CI 1.00–4.18; *p* = 0.048). See Figure 5.

## 4. Discussion

The seroprevalence studies that have been conducted in Germany so far showed a range of seroprevalence from 0.7% to 4.2% [12,13,55,56] for cats with unknown status of human infection in the household. In the presented study, only animals from known SARS-CoV-2-positive households were included, yielding a markedly higher prevalence of seropositive cats (41.7%) and a very high prevalence for dogs (55.9%). This supports earlier findings that natural interspecies transmission between humans and their pet animals occurs on a regular basis [3]. In various countries worldwide, a similar pattern was observed, with prevalences of animals from households with an unknown SARS-CoV-2 status being considerably lower than those of animals from households with known SARS-CoV-2 infections in human household members [11,14,15,17,57,58,59]. This strengthens the theory that the household is the main source of infection for pet animals.

The true prevalences found in this study for cats and dogs from infected households are in accordance with results from other, similar studies [14,60], although there are also studies that report markedly lower prevalences [58,61]. In contrast to experimental studies that indicate a higher susceptibility of cats compared to dogs [6] and other studies in infected households [60,61], we found higher prevalences in dogs than in cats. We attribute the latter on the one hand to a more intense contact between dogs and humans if dogs are kept as pets as compared to an experimental setting. On the other hand, our study involved samples from a time interval with dominance of the delta variant (between September and December 2021), whereas other studies in households [60,61] involved samples from a much wider time interval starting as early as mid-2020 and covering several variants. However, relative susceptibility of dogs and cats might vary with the virus variant.

A potential source of bias with the risk of an overestimation of seroprevalences in companion animals is the fact that nearly all households that participated (*n* = 176, 99.4%) stated that the infected persons experienced symptoms typical of a SARS-CoV-2 infection. The latter may have fostered the interest in potential transmission to dogs and cats of the household and thus, participation in the study. As the currently estimated general manifestation index in the human population is approximately 55–85% [62,63,64], an overrepresentation of symptomatic cases seems likely. Moreover, the frequency of the reported symptoms exceeded that found by the Robert Koch Institute (RKI), the German federal institute for public health. For example, coughing was reported by 84% of households, while the RKI found a proportion of 42% in the German population [65]. Furthermore, fever, which is an indicator for a more severe course of infection was reported in 55% of households of the presented study and only in 19% of German cases [65]. Asymptomatic infected humans seemed to be less likely to spread the infection [62] to other persons due to the lower virus load that was shed. Therefore, pets living in a household with symptomatic infected humans might be at a higher risk of becoming infected.

For both dogs and cats, the multivariable logistic regression model identified factors that influence the risk of acquiring an infection for the animal in question. Both analyses showed that the conditions under which an animal is kept during quarantine influence the probability of acquiring an infection, while biological factors such as having a chronic disease seem to play a less important role. For cats, the most influential factor was the number of infected humans in the household. Infected humans shed the virus while breathing [66] in the form of fine aerosols and droplets of various sizes that carry the virus [67]. Aerosols consist of tiny water particles that are less than 10 µm in diameter and can stay suspended for hours in the atmosphere [68]. The viral load in the microenvironment of the household increases with the number of infected humans present [69]. Therefore, the infectious pressure on the cat was higher if more infected humans were present.

Interestingly, in contrast to cats, for dogs, the number of infected persons in the household had no significant effect on the probability of positive test results and was excluded as an influential factor upon model optimization. The reason for this may be found in the different social role dogs and cats typically take on in a household. Dogs, as a pack animal, are keen to have at least regular contact with each member of their pack [70], while cats, as solitary hunters, choose the amount of time spent with each human household member [71]. Thus, for dogs, the effect of an increase in the infectious pressure in the environment with the number of infected humans might be masked by the dominant effect of close interaction of a dog with each household member and an increased probability of picking up the infection from single infected humans as compared to cats. However, the number of infected household members was demonstrated to be a significant risk factor for both, cats and dogs, in a study from the Netherlands [61].

The generally closer contact of dogs with household members also gives room for a marked reduction of contact during human quarantine, which possibly explains why contact reduction turns out to be a preventive factor in dogs, in contrast to cats. On the contrary, the less intense baseline contact (contact independent of the knowledge of human infection in the household) between cats and household members reduces the options for further contact reduction during quarantine, which thus has no significant effect in cats, but may explain why a baseline contact beyond average indicated by owners is a significant risk factor for cats. In a similar study conducted with a smaller sample size, the same trend of contact reduction acting as a preventive measure [60] was detected. Moreover, increased interaction between the animal and the owner was linked to an increase in the likelihood of the animal becoming infected [60,72]. However, in both studies, the effect was tested in conjunction for cats and dogs and not separately as we did in the present study. Generally, in the light of contact intensity being an important risk factor for interspecies transmission, the insight that lockdown measures lead to a higher frequency of interactions between humans and their pets [73] seems troublesome. It is crucial to highlight the importance of the reduction of direct contact between pet animals and SARS-CoV-2 infected humans and therefore, promote the establishment of hygiene rules in the handling of animals while one is infected. Such guidelines were published by various institutions [74,75,76].

Interestingly, the factor ‘contact with humans outside the infected household’ turned out to have a significant effect on the probability of positive test results in dogs, thus being a risk factor, whereas, in cats, there is a trend for this factor to act protectively. For cats, these human contacts outside the household could refer to time spent outside the household due to the SARS-CoV-2 infection of the owners or in neighbors’ gardens or houses where they were possibly even fed, thus reducing the time under risk in the environment of the original household and hence becoming a protective factor. The protective, though the not significant, effect of unsupervised outdoor access of cats is possibly based on the same principle. On the contrary, concerning dogs, contacts with humans outside the infected household will rather represent additional contacts with humans that add to the baseline risk in the household, i.e., when dogs are walked by household members and interact, for example, with other dog owners who might be infected with SARS-CoV-2. Dogs tend to have intensive close contact with other dogs and humans they meet on their walks [77], which may pose a potential risk of transmission [78].

In households with more than one participating seropositive pet, animals were significantly more likely to live with another SARS-CoV-2-positive cat or dog. This could either be due to similar circumstances under which the animals were kept within the household that favored the occurrence of interspecies transmission or due to a transmission occurring between the pets of the household. The fact that most cats and dogs that remained seronegative in a household with at least one seropositive animal were those for which a below-average contact intensity with humans was reported hints at human-to-animal transmission as the main route of infection. Nevertheless, the possibility of transmission between cats has been shown under experimental conditions [79]. Therefore, further studies are needed to explore the transmission dynamics of SARS-CoV-2 in a multiple-pet household, also if cats, as well as dogs, are kept in the same household.

Though clinical signs of animals that might have been related to infection were reported by some owners, no significant association with seropositivity could be demonstrated in our study. The latter is in line with most case reports of SARS-CoV-2 infections in cats and dogs, which describe a subclinical or mild course of disease [80,81,82,83,84,85], and findings of a comparable study from the Netherlands [61]. Furthermore, a systematic review found that the majority of cats infected with SARS-CoV-2 do not show any or only mild clinical signs [86]. Slightly more severe courses of infections, such as pneumonia, have been reported occasionally for animals with pre-existing illnesses [87]. However, an assessment of the causes of death of companion animals with laboratory-confirmed SARS-CoV-2-infection employing a patho-epidemiological model concluded that in most cases, the infection was not the primary factor for death or the decision for euthanasia, respectively [88]. Therefore, it may be concluded that dogs and cats that are infected with SARS-CoV-2 do not usually develop a disease that resembles the COVID-19 of humans.

Our study was based on samples from households in which human infection occurred between September and December 2021, when the delta variant was predominant in Germany [89]. Transmissibility to and pathogenicity in cats and dogs may be different for the omicron or other variants.

## 5. Conclusions

The presented study demonstrates that interspecies transmission of SARS-CoV-2 between humans and their pet animals occurs on a regular basis. Infected cats and dogs usually do not display clinical signs that can be observed by owners. An important risk factor for infection is, as expected, the intensity and frequency of contact at the interface of humans and pets. Therefore, the implementation of basic hygiene measurements while interacting with cats or dogs during infection with SARS-CoV-2 is strongly advised to avoid mutual infections.

## Figures and Tables

**Table 1:** Descriptive statistics of parameters.

	Cats				Dogs			
	Positive		Negative		Positive		Negative	
Number of participants	48		67		95		75	
	**Mean**	sd *	**Mean**	**sd**	**Mean**	**sd**	**Mean**	**sd**
Age (years)	6.2	5.5	6.3	5.4	6.8	3.9	5.2	4.1
Infected humans (n)	2.4	1.2	1.8	0.9	2.1	1.1	1.8	1.0
	**n**	prop. **	**n**	**prop.**	**n**	**prop.**	**n**	**prop.**
Male	30	62.5	39	58.2	45	47.4	30	40.0
One or more chronic diseases	10	20.8	16	23.9	29	30.5	18	24.0
Unsupervised outdoor access	14	29.2	32	47.8	21	22.1	12	16.0
Contact outside the household	3	6.3	12	17.9	35	36.8	17	22.7
Above-average contact intensity	32	66.7	31	46.3	57	60.0	35	46.7
Reduced contact	12	25.0	22	32.8	20	21.1	28	37.3

* standard deviation, ** proportion (%).

**Table 2:** Descriptive statistics of observed clinical signs in participating animals.

	Cats				Dogs			
	Positive		Negative		Positive		Negative	
Number of participants	48		67		95		75	
	**n**	prop. *	**n**	**prop.**	**n**	**prop.**	**n**	**prop.**
Overall symptoms	22	45.8	30	44.8	32	33.7	35	46.7
Respiratory symptoms	15	31.3	19	28.6	17	17.9	11	14.7
Reduced general health	15	31.3	19	28.6	22	23.2	18	24.0
Diarrhea	5	10.4	11	16.4	12	12.6	21	28.0

* proportion (%).

**Table 3:** Univariable analysis of parameters.

		Cats			Dogs		
Variable x_i_	Coding of x_i_	OR *	95% CI **	*p*-Value	OR	95% CI	*p*-Value
Age (years)	≤ 6 = 0, > 6 = 1	1.13	0.48–2.66	0.79	1.35	0.66–2.74	0.41
Infected humans (n)	One = 0, > one = 1	3.08	1.11–8.54	0.03	2.15	0.95–4.84	0.07
Sex	Female = 0, male = 1	1.20	0.56–2.57	0.64	1.35	0.76–2.40	0.31
One or more chronic diseases	No = 0, yes = 1	0.84	0.34–2.04	0.70	1.39	0.66–2.92	0.38
Unsupervised outdoor access	No = 0, yes = 1	0.45	0.18–1.13	0.09	1.49	0.60–3.70	0.39
Contact outside the household	No = 0, yes = 1	0.31	0.06–1.45	0.14	1.99	0.89–4.45	0.09
Above-average contact intensity	No = 0, yes = 1	2.32	0.96–5.62	0.06	1.71	0.75–3.91	0.20
Reduced contact	No = 0, yes *** = 1	0.68	0.24–1.9	0.46	0.45	0.17–1.18	0.10

* Odds ratio; ** confidence interval of odds ratio; *** reduced or discontinued.

**Table 4:** Univariable analysis of described clinical signs in participating animals.

		Cats			Dogs		
Variable x_i_	Coding of x_i_	OR *	95% CI **	*p*-Value	OR	95% CI	*p*-Value
Overall symptoms	No = 0, yes = 1	1.04	0.45–2.40	0.92	0.58	0.25–1.32	0.20
Respiratory symptoms	No = 0, yes = 1	1.15	0.49–2.71	0.75	1.27	0.51–3.17	0.61
Reduced general health	No = 0, yes = 1	1.15	0.49–2.71	0.75	0.95	0.41–2.21	0.91
Diarrhea	No = 0, yes = 1	0.59	0.16–2.18	0.43	0.37	0.11–1.21	0.10

* Odds ratio; ** confidence interval of odds ratio.

## Data Availability

The data presented in this study are available in this article.

## Supplementary Materials

The following supporting information can be downloaded at: https://www.mdpi.com/article/10.3390/v15040837/s1, Questionnaire S1 (translation of German original): Epidemiological Questionnaire—As part of the investigation of the occurrence of SARS-CoV-2 infections in pets from COVID-19-affected households; Figure S1: Absorbance of serum samples tested with an indirect multispecies ELISA against the receptor-binding domain (RBD) of the SARS-CoV-2 spike protein.
