# Supplementary material for: SARS-CoV-2 Infection and Clinical Signs in Cats and Dogs from Confirmed Positive Households in Germany
Source: Viruses. 2023 Mar 24;15(4):837. doi: 10.3390/v15040837 (PMC10144952; doi:10.3390/v15040837)
Supplement: Supplementary file 1 [file viruses-15-00837-s001.zip › Figure S1.pdf]

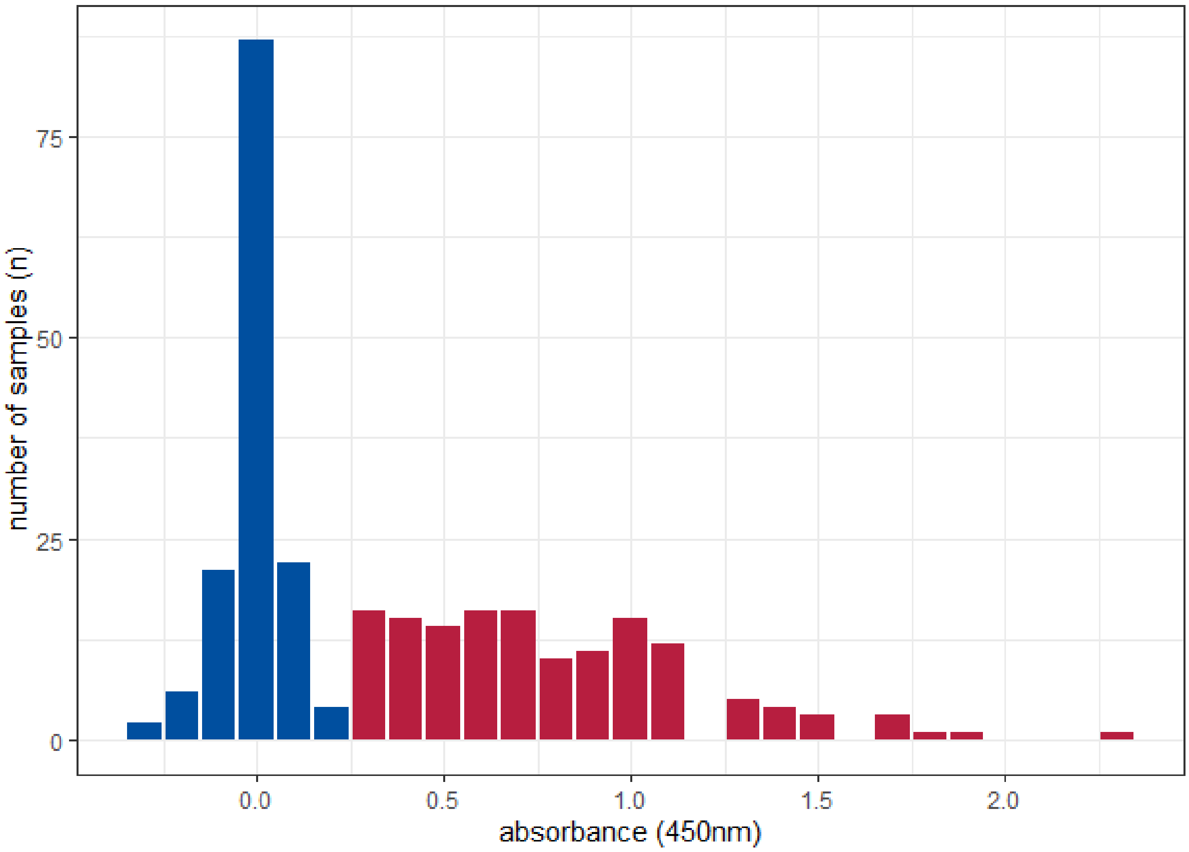

Figure S1. Absorbance of serum samples tested with an indirect multispecies ELISA against the receptor-binding domain (RBD) of the SARS-CoV-2 spike protein. The absorbance was calculated by subtracting the optical density (OD) of the uncoated well from the OD of the well coated with the RBD protein and is given on the x-axis. The y-axis shows the number samples (n) that were measured at the respective absorbance. The cut-off value is defined at an absorbance of 0.3 according to the previous test validation. Samples declared as positive are marked in red and samples declared as negative are marked in blue.
