# Supplementary material for: SARS-CoV-2 Infection and Clinical Signs in Cats and Dogs from Confirmed Positive Households in Germany
Source: Viruses. 2023 Mar 24;15(4):837. doi: 10.3390/v15040837 (PMC10144952; doi:10.3390/v15040837)
Supplement: Supplementary file 1 [file viruses-15-00837-s001.zip › Questionnaire S1.pdf]

Epidemiological Questionnaire

As part of the investigation of the occurrence of SARS-CoV-2 infections in pets from COVID-19-affected households

Friedrich-Loeffler-Institut
Federal Research Institute for Animal Health
attn. Anna Michelitsch
Südufer 10
17493 Greifswald - Insel Riems

ID-number: CoV21_xxx

Pet owner:

full name: ____________________

home address: ____________________

____

phone number.: ____________________

Email address: ____________________

1. Household details:

How many people live in the household and how many of them were infected with SARS-CoV-2?

person	age	positive test for SARS-CoV-2?	clinical symptoms?
1		☐ yes, starting on:	☐ yes, starting on:
2		☐ yes, starting on:	☐ yes, starting on:
3		☐ yes, starting on:	☐ yes, starting on:
4		☐ yes, starting on:	☐ yes, starting on:
5		☐ yes, starting on:	☐ yes, starting on:
6		☐ yes, starting on:	☐ yes, starting on:
7		☐ yes, starting on:	☐ yes, starting on:
8		☐ yes, starting on:	☐ yes, starting on:

If more space is needed, please use the backside

Which of the following symptoms of illness occurred in the household member(s) sick with COVID-19?

- ☐ cough
- ☐ disturbances in smelling and impaired sense of taste
- ☐ cold
- ☐ sore throat
- ☐ diarrhoea
- ☐ cconjunctivitis
- ☐ fever
- ☐ shortness of breath / respiratory distress
- ☐ headache
- ☐ nausea
- ☐ others: ______________________________

2. Details of the animal presented for blood collection in the context of the study:

animal species:

☐ dog ☐ cat ☐ others, namely: ____________________

breed: ____________________

age: __________

sex: __________

weight: __________

Does your pet have one or more underlying (chronic) diseases?

____

____

How long has the animal been in your possession? ____________________

Are there any other animals living in your household? If yes, what species(es)?

☐ no ☐ yes, namely: ______________________________

Does your pet have unsupervised outdoor access?

☐ no ☐ yes

Does your pet catch or eat wild small mammals, such as mice, shrews, etc.?

☐ no ☐ yes

Has your pet exhibited any of the following symptoms or behavioral changes in temporal relation to the detection of SARS-CoV-2 infection and the quarantine of residents in your household? *Please do not include symptoms of an underlying chronic disease unless they have increased in severity.*

	before the test result	during quarantine	after quarantine
cough	☐	☐	☐
nasal discharge	☐	☐	☐
labored breathing	☐	☐	☐
reduced resilience	☐	☐	☐
reduced appetite	☐	☐	☐
increased need for rest	☐	☐	☐
diarrhea	☐	☐	☐
other abnormalities (please name):			
______________________	☐	☐	☐
______________________	☐	☐	☐
______________________	☐	☐	☐
______________________	☐	☐	☐

3. Details of animal-human interaction

Which of the following statements are true about the relationship between diseased or positive SARS-CoV-2 tested household members and your animal?

	daily	several times a week	sometimes	never
I/we cuddle with the animal.	☐	☐	☐	☐
I/we kiss the animal.	☐	☐	☐	☐
I/we let the animal sniff us.	☐	☐	☐	☐
I/we let the animal lick our hands.	☐	☐	☐	☐
I/we let the animal lick our face.	☐	☐	☐	☐
My/our pet sleeps in my/our bed.	☐	☐	☐	☐
My/our pet is lying on other shared furniture (sofa, chair, etc.).	☐	☐	☐	☐
I/we feed the animal at or from the table.	☐	☐	☐	☐
After the meal, the animal may lick my/our used tableware.	☐	☐	☐	☐

Where did the animal stay during the quarantine of the household member who tested positive for SARS-CoV-2?

- ☐ in our household
- ☐ with friends or relatives
- ☐ in a boarding kennel
- ☐ in an animal shelter
- ☐ at another location (please specify): ______________________________

Did you reduce or stop contact between your pet and those in your household who tested positive for SARS-CoV-2 during the quarantine period?

☐ yes, reduced ☐ yes, discontinued ☐ no, everything remained unchanged

Did your pet have regular contact with other people outside your household (*e.g., walking service during quarantine, visits of your cat to neighbors, etc.)?*
Are you aware of infections with SARS-CoV-2 from these persons?

	type of contact	frequency (*daily, several times a week, weekly*)	SARS-CoV-2 infection known?
1			☐ yes ☐ no
2			☐ yes ☐ no
3			☐ yes ☐ no
4			☐ yes ☐ no
5			☐ yes ☐ no
6			☐ yes ☐ no
